# Shopping for others: how does inconsistency in online reviews affect purchase intentions differently?

**DOI:** 10.3389/fpsyg.2025.1579545

**Published:** 2025-06-19

**Authors:** Jiaying Wang, Yang Liu, Zeguo Qiu, Zhijie Zhao, Meng Wang, Hong Lan

**Affiliations:** ^1^School of Opto-Electronic Engineering, Zaozhuang University, Zaozhuang, China; ^2^School of Business, Quzhou University, Quzhou, China; ^3^China Postdoctoral Research Station of Northeast Asia Outsourcing Service Research Center, Harbin University of Commerce, Harbin, China; ^4^School of Computer and Information Engineering, Harbin University of Commerce, Harbin, China

**Keywords:** online review inconsistency, social distance, purchase intention, construal level theory, product type

## Abstract

**Introduction:**

The product reviews on e-commerce platforms often exhibit inconsistencies in consumer opinions. This raises the question: when consumers purchase products for others, do they place greater importance on inconsistent online reviews? To address this issue, this study explores the impact of online review consistency, social distance, and product type on consumer purchase intentions, based on the theories of psychological distance and construal level. It focuses on examining the moderating roles of social distance and product type in this process.

**Methods:**

A scenario-based experimental design was employed to test the hypotheses, with analysis of variance and simple effects tests used for data analysis. Study 1 utilized a 2 (inconsistency of online reviews: inconsistent/consistent) × 2 (social distance: close/remote) between-subjects design. Study 2 employed a 2 (inconsistency of online reviews: inconsistent/consistent) × 2 (social distance: close/remote) × 2 (product type: experience/search) design. Participants in each group were exposed to different experimental conditions and evaluations to assess the impact of online review consistency, social distance, and product type on consumer purchase intentions.

**Results:**

The study finds that inconsistency in online reviews significantly negatively impacts consumers’ purchase intentions, with ambivalent attitudes serving as a mediating factor. Under the influence of social distance matching effects, consumers exhibit lower purchase intentions when purchasing for a distant social relationship (i.e., with a remote social distance) compared to when purchasing for a close social relationship (i.e., with a close social distance), in situations involving inconsistent reviews. However, no significant difference is found in situations with consistent reviews. Product type further moderates the impact of social distance on the effect of online review inconsistency on consumers’ purchase intentions. Specifically, when consumers face inconsistent online reviews and the product recipient is a close relationship, their purchase intention for experience products (vs. search products) is lower. In contrast, when the recipient is a distant relationship, the difference in purchase intention is not significant.

**Discussion:**

The findings of this study provide support for the mechanism through which online review inconsistency affects consumer purchase intentions, offering a new perspective for future research on the impact of review inconsistency. Additionally, it provides theoretical support for businesses and companies engaged in online marketing on e-commerce platforms.

## 1 Introduction

With the advancement of information technology and the development of the internet, people’s consumption habits have quietly changed, with an increasing preference for shopping on various e-commerce platforms and sharing their reviews and opinions. The survey indicates that 93.4% of customers base their purchasing decisions on the online reviews they read (Singh et al., 2023). Online reviews contain a wealth of authentic product information, effectively reducing the risks associated with online shopping and supporting consumers’ purchase decisions. However, due to differences in product quality and consumer experiences, online reviews for the same product are often diverse and may even exhibit inconsistency (Shan et al., 2021). This inconsistency in online reviews has a certain impact on consumers’ purchase intentions. Recently, scholars have focused on how inconsistencies in online reviews affect consumers’ purchase intentions (Zhang et al., 2023) Making a purchase decision is relatively easy when all reviews are either positive or negative. However, when reviews are mixed, containing both positive and negative opinions, this inconsistency increases consumers’ cognitive load (Chen et al., 2022; Wang Y. et al., 2023) and leads to ambivalent attitudes (Natarajan and Periaiya, 2024), complicating the decision-making process (Wang J. et al., 2023).

It is important to recognize that current research often neglects a significant aspect of purchasing behavior—the variability in product recipients. In reality, when consumers engage in online shopping, they not only make purchases for themselves but frequently also buy items for others. In such scenarios, it is imperative for consumers to consider the habits and attitudes of other potential users towards the product when making their purchase decisions (Tavitiyaman et al., 2024). When buying gifts, consumers exhibit even greater caution, dedicating more time and effort to select products that cater to the preferences of the intended recipients (Wooten, 2000). Moreau and colleagues argue that consumers feel they invest more time and thought in customizing products for others than for themselves and are also more inclined to spend additional money (Moreau et al., 2011). Additionally, Baskin and associates have noted that during the gift-buying process, consumers prioritize whether the gift will be well-received over its practical utility (Baskin et al., 2014). Previous studies have demonstrated that the impact of online reviews on consumers’ purchasing intentions varies significantly across different product types (Liu et al., 2020). Therefore, when purchasing diverse types of products online, such as experiential goods like dining out or functional goods like office equipment, it is essential for consumers to consider the preferences of all involved parties. The question arises: Does this consideration vary depending on the product type?

**Figure 1:**
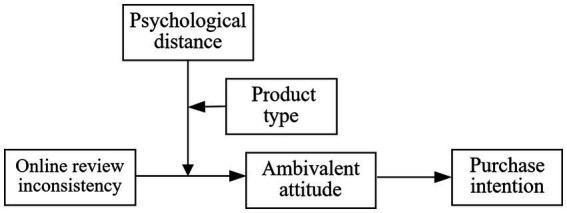
Model of the mechanism whereby inconsistency of online reviews affects consumer purchase intention.

The current study, based on the Construal Level Theory, aims to explore how the inconsistency of online reviews affects consumers’ purchase intentions from the perspective of social distance, focusing on ambivalent attitudes as a key mediator. The research framework, as illustrated in Figure 1, intends to uncover the mechanisms through which the inconsistency of online reviews influences consumers, focusing on three key questions:

1. How does the inconsistency of online reviews influence consumers’ purchase intentions through the mediating role of ambivalent attitudes?
2. How does social distance moderate (a) the relationship between online review inconsistency and ambivalent attitudes, and (b) the subsequent impact of ambivalent attitudes on purchase intentions?
3. How does product type (search vs. experience) moderate the interplay between psychological distance and online review inconsistency in shaping purchase intentions?

## 2 Theories and hypotheses

### 2.1 Construal level theory and psychological distance theory

Construal Level Theory (CLT) is a social cognitive theory that highlights individuals’ perception and understanding of phenomena. Consequently, individuals’ psychological representations of phenomena influence their reactions when encountering corresponding stimuli. However, due to variations in the characteristics of the perceptual process, individuals develop different psychological representations when facing different stimuli. According to CLT, individuals’ perception of phenomena typically occurs hierarchically, constituting a continuous process (Ram et al., 2024). This perception categorizes into high and low construal levels based on different representational modes (Li et al., 2009). As research has progressed, scholars have incorporated CLT into the analysis of psychological distance, gradually evolving into Psychological Distance Theory. This theory posits that psychological distance, referenced to the individual themselves, subjectively perceives when and where events occur, who they involve, and the likelihood of event occurrences (Huang et al., 2015; Liberman and Trope, 1998). It represents the subjective intensity of an individual’s perception of external events (Maglio, 2020). CLT suggests that individuals’ interpretation of future objective events is influenced by temporal distance, subsequently affecting their judgments and behaviors (Zheng et al., 2023). Research on the various dimensions of psychological distance has gradually commenced, driving the ongoing advancement of CLT. Trope and Liberman have constructed a unified psychological distance framework comprising social distance, spatial distance, temporal distance, and hypotheticality. They have also proposed and refined Construal Level Theory (Liberman et al., 2002; Liberman and Trope, 1998; Trope and Liberman, 2010). Under high construal levels, consumers demonstrate heightened attention to abstract informational dimensions in online reviews, including source credibility, holistic evaluations, and value congruence. Conversely, at low construal levels, cognitive processing prioritizes concrete product specifics such as functional attributes, usage scenarios, and risk-related negative disclosures—characteristics aligned with utilitarian decision-making frameworks.

On one hand, psychological distance affects construal level, indirectly influencing individuals’ behavioral intentions. Typically, when psychological distance is greater, individuals use a high construal level to understand events, emphasizing simple and essential characteristics (Weidlich et al., 2024). Conversely, when psychological distance is shorter, individuals use a low construal level to understand events, focusing on contextualized and superficial characteristics. On the other hand, construal level can reciprocally affect psychological distance, influencing individuals’ perception of psychological distance. Generally, when faced with events that are psychologically distant, individuals tend to interpret them using a high construal level. Conversely, when faced with events that are psychologically near, individuals tend to interpret them using a low construal level (Bar-Anan et al., 2006). High construal levels are more common in interpreting events in daily life. When explaining people’s consumer behavior, individuals often refer to their distant past, distant spatial locations, advice from others at a distance in social relationships, or purchase decisions in high probability situations. These simple and essential mental representations have high reference value for similar events (Ram et al., 2024). Therefore, to a certain extent, different construal levels can constrain an individual’s psychological perception while also expanding it. For example, building upon Construal Level Theory, Smith and Trope demonstrated that social distance – operationalized as individuals’ perceived similarity to or intimacy with social targets – systematically activates differential information processing mechanisms (Smith and Trope, 2006). To illustrate, in gift-giving scenarios involving socially distant recipients (e.g., casual colleagues), which represent high social distance contexts, abstract review attributes such as source credibility predominantly engage high-level construal cognition, thereby exerting stronger influence on purchase intentions. Conversely, when selecting gifts for socially proximal recipients (e.g., close friends) in low social distance situations, concrete review characteristics including informational specificity and textual readability predominantly activate low-level construal processing, which more substantially impacts decision-making outcomes (Chatterjee, 2023).

In summary, there is a mutual influence between psychological distance and construal level, grounded in the events and individual experiences (Wakslak and Joshi, 2020). When there is a significant disparity between events and individual experiences, individuals perceive the events at a greater psychological distance. In such cases, they struggle to obtain detailed information about the events, leading to a tendency towards simplistic interpretations, resulting in a high construal level. Conversely, when the gap between events and individual experiences is smaller, individuals perceive the events at a closer psychological distance. They can easily acquire information about the events, leading to a tendency towards specific interpretations, resulting in a low construal level. On the other hand, when events are perceived as simple, individuals perceive them at a greater psychological distance. Conversely, when events are perceived as detailed and complex, individuals perceive them at a closer psychological distance (Zhu et al., 2011).

### 2.2 Online review inconsistency

Online review inconsistency sparks users’ exploratory interests, leading them to pay closer attention and perceive the reviews as more objective and genuine. The higher the online review inconsistency, the greater its impact on users’ purchase decisions (Purnawirawan et al., 2015). Shi et al. examined the consistency of initial and follow-up reviews and pointed out that follow-up reviews with no fundamental change in attitude fail to capture audience attention. However, differences in attitude changes can influence audience purchasing decisions, with consumers preferring to rely on follow-up reviews for judgment and selection (Shi et al., 2016). Tang et al.’s research suggests that inconsistent reviews imply product instability in quality, reducing consumers’ willingness to purchase and thus harming product sales (Tang et al., 2014). Generally, consistent information is considered more credible than inconsistent information (Cialdini, 2001). Compared to inconsistent online reviews, consistent ones maintain the same evaluative stance throughout, enabling consumers to easily and directly discern the reviewers’ attitudes. As a result, consumers typically opt for consistent information for analysis and judgment (Jun Wang and Zhou, 2016). Zhu and Zhang’s study indicates that consumers exhibit stronger purchase intentions when encountering products with consistent online reviews (Zhu and Zhang, 2010). In traditional online review studies, consensus reviews, where multiple reviewers express similar viewpoints, are deemed more trustworthy than inconsistent ones (Lo and Yao, 2019; Zhao et al., 2018). Therefore, we hypothesize:

*H1*: Online Review Inconsistency has a significant negative impact on consumer purchase intention.

Existing literature suggests that inconsistency in information increases consumers’ cognitive load (Wang Y. et al., 2023) and can lead to conflicting attitudes in individuals (Natarajan and Periaiya, 2024). According to the Cognitive Consistency Theory (Gawronski and Strack, 2012), consumers encountering inconsistent review information online may develop conflicting attitudes, especially when they cannot resolve this attitude discrepancy through information gathering (Conner and Sparks, 2002). This may decrease their willingness to purchase the product. A recognized outcome of conflicting attitudes is its impact on attitude-behavior consistency (Yang and Unnava, 2016). Studies suggest that conflicting attitudes lead to consumers exhibiting indecisive, unstable, uncertain, and directionless behaviors toward products (Conner et al., 2002; Yang and Unnava, 2016). Gursoy’s pioneering discussion suggests that ambiguous and inconsistent information can result in consumers being unable to accurately process information about hotel attributes (Gursoy, 2019).

Therefore, when consumers encounter online review inconsistency while shopping online, they may struggle to extract valuable information from the reviews, leading to heightened levels of ambivalent attitude. The characteristics of uncertainty and lack of direction inherent in inconsistent reviews can further exacerbate consumers’ confusion. Ambivalent attitudes refer to a cognitive conflict state in which an individual holds both positive and negative evaluations towards a single target object simultaneously (Jonas et al., 2000). Penz and Hogg suggest that ambivalent attitudes mediate between physical, market, and personal factors influencing consumers’ purchase intentions (Penz and Hogg, 2011). Personal factors, such as the consumer’s closeness to the recipient of the product, also play a role in the formation of ambivalent attitudes. When consumers purchase products for individuals with whom they have a close relationship, their evaluation of the product tends to be more subjective, and ambivalent attitudes may be lower. In contrast, when purchasing products required by others, consumers are more likely to consider social distance, leading to higher ambivalent attitudes. Similarly, scholars have found that stronger ambivalent attitudes lead consumers to abandon purchasing products (Moody et al., 2014). Additionally, Xie et al. affirmed that exposure to online review inconsistency undermines consumers’ decision predispositions and weakens their intentions (Xie et al., 2011). Hence, we hypothesize that in the online shopping environment, the ambivalence generated by online review inconsistency may result in diminished purchase intentions among consumers. Drawing upon the construal level theory (Gawronski and Strack, 2012), this study suggests that compared to consistent online reviews, consumers exposed to online review inconsistency may develop ambivalent attitudes towards the product, thereby reducing purchase intentions. Thus, we posit that ambivalent attitudes mediate the relationship between online review inconsistency and purchase intentions (Siddiqi and Akhtar, 2021). Therefore, we hypothesize:

*H2*: Ambivalent attitudes mediate the relationship between online review inconsistency and purchase intentions.

### 2.3 Moderating effect of social distance

Social distance refers to the degree of closeness an individual subjectively desires to establish with others, representing the perceived proximity between an individual and certain people. Different types of relationships between individuals allow people to distinguish the perceived social distance from one another. Therefore, close social distance is the psychological distance formed when individuals are in a state of intimate social relationships or similar identities, while remote social distance refers to the opposite. Construal Level Theory (CLT) suggests that, compared to negative information, the influence of positive information increases as psychological distance increases (Trope and Liberman, 2010). The CLT posits that individuals predict negative information when making judgments, thereby explaining the mechanisms by which individuals process and evaluate different information (Trope and Liberman, 2010). In contrast to negative information, the impact of positive information increases with psychological distance. Evidence supporting this reasoning demonstrates that as participants make judgments about entities that are socially distant, the likelihood of positive actions significantly increases with the increase in social distance (Kyu Kim et al., 2021). Moreover, because abstract information enhances the positivity of both negative and positive emotional experiences, individuals seem to evaluate negative and positive emotional experiences more positively when engaging in high-level construals (Williams et al., 2014). In such instances, when faced with inconsistent information, individuals also pay more attention to positive information and make more positive decisions as social distance increases.

Consumers often make purchases not only for themselves but also frequently for others, necessitating the consideration of others’ product attitudes in their purchase decisions. For instance, when buying furniture or deciding where to dine, consumers need to take into account the preferences of other co-decision-makers (Krishnamurthi, 1988); when purchasing products for others (such as selecting gifts), consumers especially need to invest effort in catering to the preferences of the recipients (Wooten, 2000). Scholars have investigated the divergence in consumers’ key considerations during product acquisition for recipients across different social ties, examining this phenomenon through diverse analytical lenses (Yan and Sengupta, 2011). Moreau et al. demonstrated that consumers perceive greater expenditure of effort and time when customizing products for close ties (vs. remote ties), thereby exhibiting heightened willingness to pay a price premium (Moreau et al., 2011).

When consumers shop online, under conditions of remote social distance, such as when purchasing products for individuals with whom they have a remote relationship, they activate a low construal level. In this scenario, detailed product information in online reviews has a greater influence on them (Wang J. et al., 2023). Therefore, inconsistencies in online reviews have a stronger impact on consumers’ ambivalent attitudes and purchase intentions. However, under conditions of close social distance, such as when purchasing products for individuals with whom they have a close relationship, consumers activate a high construal level and pay more attention to the abstract information in product reviews (Wang J. et al., 2023). Compared to remote social distance, the influence of online review inconsistency on consumers’ ambivalent attitudes and purchase intentions is weaker. Based on this, the following hypotheses are proposed:

*H3*: Social distance moderates the impact of online review inconsistency on consumers’ ambivalent attitudes, thereby affecting their purchase intentions.

*H3a*: Faced with inconsistent online reviews, consumers exhibit lower purchase intentions in the remote social distance (vs. close social distance) condition.

*H3b*: Faced with consistent online reviews, consumers exhibit lower purchase intentions in the close social distance (vs. remote social distance) condition.

### 2.4 The second-order moderating effect of product type on social distance

Nelson categorizes products into search and experience goods from the perspective of information economics (Nelson, 1974). In terms of shopping practices, search goods are relatively easy to obtain quality information about before purchase, whereas experience goods are not. Compared to experience goods, consumers conduct more searches when purchasing search goods. Information about search goods is easily displayed objectively, while information about experience goods tends to be subjective. Research indicates that online reviews on e-commerce platforms have different impacts on consumers’ willingness to purchase online across different product types (Zhang and Li, 2016). Generally, the quality and performance information of search goods can be reflected in objective data, such as computers, smartphones, routers, etc. On the other hand, the quality and performance of experience goods require subjective experiences and feelings from consumers to know, making it difficult to express in objective data, such as food, clothing, cosmetics, etc. Different types of products have varying influences on consumers’ purchase decisions; experience goods provide consumers with more experiences and services, while search goods focus more on the intrinsic value of the product itself (Zhang and Li, 2016). The moderating effect of product type on the relationship between social distance and consumer purchase decisions can be explained using the interaction between construal level theory and product attribute classification (Wang and Zhou, 2016). The quality of search products can be assessed in advance through objective parameters, whereas the quality of experience products heavily relies on subjective feelings. When consumers are in a situation of close social distance, low construal-level cognition is activated, and the influence of social distance on search products becomes more significant (Wang J. et al., 2023). In this context, the quality information of the product is crucial, and the decision-making process depends more on rational analysis. In contrast, when consumers are in a situation of remote social distance, they tend to adopt high-level, decontextualized interpretations. At this point, the influence of social distance is greater for experience products, and consumers need to rely on others’ consumption experiences to reduce cognitive uncertainty. Therefore, we argue that the emphasis of review information that consumers pay attention to when purchasing different types of products online varies. Consumers tend to focus on objective evaluations when buying search goods, whereas they pay more attention to subjective evaluations with emotional inclinations when purchasing experience goods (Zhang and Li, 2016). Inconsistencies in online reviews have different effects on consumers across different product types, thus leading to variations in consumers’ conflicting attitudes.

In the hypotheses proposed in this study, social distance serves as a moderating variable. In the actual context of online shopping, consumers perceive different levels of social distance due to the recipients of the purchased products, leading to varying levels of perceived social distance. Additionally, the focus of consumers’ purchase decisions varies when buying different types of products. Therefore, it is necessary to examine the interaction between these two moderating variables and their impact on consumer purchase intentions. Research indicates that product type also influences the way recommenders disseminate information, thereby affecting the recipients’ discerning thinking (Huang et al., 2018). Moreover, the alignment between product type and construal level can effectively moderate the relationship between consumer perception and evaluation (Huang et al., 2018). We posit that product type has a certain impact on consumers’ perception of social distance. Under the same social distance condition, the effect of inconsistent online reviews is more pronounced in search products, leading to a higher level of conflicting attitudes and lower purchase intentions among consumers (Zhang and Li, 2016). Considering the moderating effect of social distance, we propose the following second-order moderating hypotheses regarding product type:

*H4*: Product type further moderates the relationship between social distance and the impact of inconsistent online reviews on consumer ambivalence, subsequently influencing purchase intentions.

*H4a*: In the presence of inconsistent online reviews, under conditions of close social proximity, consumers exhibit lower purchase intentions for experiential products compared to search products. Conversely, under conditions of remote social proximity, their purchase intentions are higher.

*H4b*: In the presence of consistent online reviews, under conditions of remote social proximity, consumers exhibit higher purchase intentions for experiential products compared to search products. Conversely, under conditions of close social proximity, their purchase intentions are lower.

We have established a research model as shown in Figure 1.

## 3 Research methodology

### 3.1 Study 1

Study1 employed a 2 (inconsistency of online reviews: inconsistent/consistent) × 2 (social distance: close/remote) between-subjects experiment. Firstly, it aimed to validate the impact of inconsistent online reviews on consumers’ purchase intentions and whether it operates through cognitive dissonance, thus examining the main effect, i.e., testing hypothesis H1. Secondly, it aimed to assess whether different levels of social distance act as moderators, thus verifying hypothesis H2.

#### 3.1.1 Research process

The experimental product in this study should possess both experiential and search characteristics. Bluetooth headphones were ultimately selected as a representative product with both search and experience features. Bluetooth headphones exhibit characteristics of search products, where consumers can evaluate the quality of the product through objective performance metrics such as effective range and talk time. Simultaneously, they also possess features of experience products, where consumers need to use the product before making judgments, such as comfort in wearing. This aspect has also been validated in previous research. Qiu Lingyun, in a study on the usefulness of online reviews, used headphones as representatives of experience products (Qiu et al., 2019), while Huang Jing used Bluetooth headphones as representatives of search products in a study on the impact of online image presentation order on consumers’ purchase intentions (Huang et al., 2016). Therefore, previous studies have adequately demonstrated that Bluetooth headphones possess both search and experience characteristics. We chose Bluetooth headphones as representatives of products with both search and experience features. The questionnaire in this study utilized a 7-point Likert scale, with responses scored from 1 to 7 (Ahmad et al., 2023).

The subject of this study is the inconsistency of online reviews for products on e-commerce platforms. Online reviews were sourced from Taobao, which is the largest B2C shopping website in China and has abundant data (Shi et al., 2018). Therefore, this study chose to filter online review data for Bluetooth headphones from Taobao. Brand information for Bluetooth headphones was concealed in the context experiment to prevent brand factors from influencing the experimental results (Keller and Lehmann, 2006). This experiment drew on the method of Chen and Lurie to obtain negative online reviews. Initially, positive reviews for products were obtained from the e-commerce platform. Then, words with positive polarity in the positive reviews were replaced with antonyms or negated by adding negations, thus generating negative reviews. This effectively avoided the influence of factors such as language style and word count on the participants (Chen and Lurie, 2013; Wang et al., 2015). For example, a positive online review stating “the sound quality is excellent, and the touch is very sensitive” was modified into a negative online review stating “the sound quality is not very good, and the touch is not sensitive enough.”

In previous experimental research in the field of consumer decision-making, college student samples are often used for initial theory testing before broader validation (Huang et al., 2018). The participants in this study were college students from a university in Heilongjiang Province. College students were chosen as participants because they typically have extensive online shopping experience. Using the same sample type reduces variability and minimizes the influence of irrelevant factors on the experimental study, thereby enhancing the internal validity of the research (Peterson, 2001). A total of 288 participants were recruited for this study, and they were randomly assigned to four experimental conditions in different time slots. Differential manipulation was conducted during the experiment. For instance, in the close social distance condition, the scenario described was: “Your closest friend’s Bluetooth headphones recently broke, and you plan to buy a new pair for him (her). You noticed a certain brand of Bluetooth headphones on a shopping website. Please first review the textual and visual information about these Bluetooth headphones.” In the remote social distance condition, the scenario described was: “Your university class is organizing an event, and a classmate (with whom you have an ordinary relationship) lacks Bluetooth headphones. As the person in charge of the event, you are tasked with selecting headphones for him (her). You noticed a certain brand of Bluetooth headphones on a shopping website. Please first review the textual and visual information about these Bluetooth headphones.” The relevant textual and visual information about the Bluetooth headphones is provided in Appendix 1. Participants then completed the social distance manipulation check items, where they were asked to indicate their perception of the social distance described in the materials based on their own feelings, as shown in the first question of Appendix 1. For example, in the remote social distance condition, participants were asked: “Compared to your ‘closest friend,’ how distant do you perceive the ‘ordinary classmate’ described in the materials to be?”

Participants were then asked to complete the item measuring perceived inconsistency in online reviews based on their own feelings, as shown in the second question of Appendix 1. They then filled out the scale items corresponding to ambivalent attitudes, with the scores representing the participants’ ambivalent attitudes, as shown in the third question of Appendix 1. Finally, participants completed the purchase intention scale to measure their purchase intention, as shown in the fourth question of Appendix 1.

Next, before starting to read the online reviews, there was a situational instruction: “Before purchasing Bluetooth headphones, you want to understand the online reviews of consumers who have purchased this product before making a decision. Please carefully read the relevant reviews and then select your answers to the questions in the questionnaire.” The inconsistency of online reviews was manipulated, with participants in different groups reading different types of online reviews. Participants in the inconsistent group read inconsistent online reviews, while those in the consistent group read consistent online reviews.

Subsequently, participants were asked to make perceptual judgments about the inconsistency of online reviews based on their own feelings. They then filled out items on a measurement scale corresponding to ambivalent attitudes, with the scores indicating consumers’ ambivalent attitudes. Next, they completed a purchase intention scale to measure their purchase intentions. Finally, measurements were taken for control variables in this study, namely participants’ basic information (gender, online shopping experience, disposable income, duration of online shopping). After the completion of the situational experiment, all participants will receive a high-quality souvenir worth $3.50 as a token of appreciation.

The questionnaires were reviewed, and incomplete responses as well as those from participants who had never engaged in online shopping were excluded. In total, 284 valid questionnaires were collected, resulting in a response rate of 98.6%. Based on calculations from previous research, the data volume meets adequacy requirements (Irshad et al., 2024). The statistics revealed that among the participants, there were 132 males, accounting for 46.5%, and 152 females, accounting for 53.5%, which satisfied the gender requirements of the experiment. Regarding online shopping experience, 37 participants had purchased items online a few times, 209 participants frequently shopped online, and 38 participants preferred to buy items online whenever possible, indicating that the majority of participants were regular online shoppers. In terms of disposable monthly income, 45 participants had incomes below 1,000 yuan, 183 participants had incomes ranging from 1,001 to 2000 yuan, and 56 participants had incomes exceeding 2001 yuan. Regarding the duration of online shopping, 22 participants had been shopping online for less than 1 year, 95 for 1–3 years, 127 for 3–6 years, and 40 for over 6 years. The demographic characteristics of the participants aligned with the actual situation, meeting the requirements of this study.

#### 3.1.2 Results

##### 3.1.2.1 Manipulation check

Social Distance Assessment: Drawing from previous situational experiment manipulation experiences, participants’ perceptions of social distance were measured. Among the 284 valid participants who underwent manipulation, 142 were in the close social distance condition, and 142 were in the remote social distance condition. Statistical analysis was conducted to examine the difference in social distance perceived between purchasing for the closest friend and purchasing for an ordinary classmate. Purchasing for the closest friend was categorized as close social distance (*M* = 3.201, SD = 1.4789), while purchasing for an ordinary classmate was categorized as remote social distance (*M* = 4.236, SD = 1.3329). The difference in social distance between them was significant (*F* = 43.550, *p* = 0.000﹤0.05), indicating successful manipulation of social distance.

Inconsistency of Online Reviews Assessment: Lower scores on the items assessing perceived inconsistency of online reviews indicate a stronger perception of inconsistency by consumers. Participants in the inconsistent group (*M* = 3.7355, SD = 1.0736) perceived a stronger inconsistency in reviews compared to those in the consistent group (*M* = 4.4822, SD = 1.1402) (*F* = 1.237, *p* = 0.000 < 0.05), demonstrating successful manipulation of inconsistency of online reviews.

##### 3.1.2.2 Mediation analysis

This study employed Bootstrap to analyze the mediating effect of ambivalence attitude, with the independent variable (X) being inconsistency of online reviews, the mediator (M) being ambivalence attitude, and the dependent variable (Y) being purchase intention. Model 4 of Bootstrap was chosen with a self-sampling sample size of 5,000 and a 95% confidence interval.

**Figure 2:**
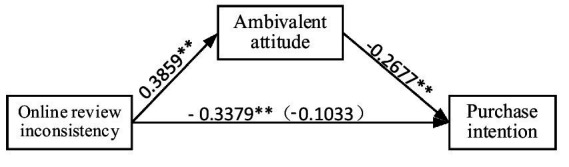
Testing the mediating role of ambivalent attitude.

(1) Analysis of the mediation effect of consumer ambivalence attitude (M): As shown in Figure 2, online review inconsistency has a negative impact on consumer purchase intention (−0.3379), with a significance level less than 0.01. This result aligns with Research Hypothesis H1; therefore, Hypothesis H1 is supported. The independent variable inconsistency of online reviews (X) has an indirect effect on the dependent variable consumer purchase intention (Y). The analysis results of BOOTLLCI and BOOTULCI for the mediator, consumer ambivalence attitude (M), were-0.2615 and-0.0075, respectively, neither of which includes 0. This indicates the existence of the mediating effect of consumer ambivalence attitude (M), with a magnitude of-0.1033. Therefore, inconsistency of online reviews can influence consumer purchase intention through the mediating effect of consumer ambivalence attitude (M), with the mediating effect (−0.1033) accounting for 23.4% of the total effect. The next step was to determine whether the mediating effect of consumer ambivalence attitude (M) was partial or full mediation. Examining the direct effect of the independent variable inconsistency of online reviews (X) on the dependent variable consumer purchase intention (Y), the values of LLCI and ULCI were-0.6257 and-0.0501, respectively, neither of which includes 0. This indicates that the mediating effect of consumer ambivalence attitude (M) is partial mediation, with this direct effect (−0.3379) accounting for 76.6% of the total effect.

(2) Analysis of the effect of consumer ambivalence attitude on purchase intention: The regression analysis results of the joint effect of the independent variable inconsistency of online reviews (X) and the mediator variable consumer ambivalence attitude (M) on consumer purchase intention (Y) indicate that consumer ambivalence attitude (B) has a significant regression effect on the dependent variable consumer purchase intention (Y), with LLCI and ULCI values of –0.4143 and –0.1211, respectively, neither of which includes 0. This indicates that consumer ambivalence attitude has a significant negative impact on purchase intention, with a coefficient of –0.2677. Therefore, consumer ambivalence attitude has a significant negative impact on purchase intention, which aligns with the hypothesis of this study. Hypothesis H2 is supported and validated. For detailed test results, refer to Figure 2.

##### 3.1.2.3 Analysis of social distance regulation effect

In order to delve deeper into the factors influencing consumer purchase intentions, we investigate the interactive effect of online review inconsistency and social distance on consumer purchase intentions to validate the moderating effect of social distance on the impact of online review inconsistency on purchase intentions. To examine the moderation effect of psychological distance, between-group variance analysis was conducted on different groups. Online review inconsistency (inconsistent/consistent) was treated as the independent variable, social distance (close/remote) as the moderating variable, and consumer purchase intentions as the dependent variable for the analysis of variance. Detailed experimental analysis results are presented in Table 1.

**Table 1:** Between-group variance analysis (dependent variable: purchase intentions).

Source	III Type sum of squares	Degrees of freedom	Mean square	*F*	Significance
Corrected model	29.814^a^	3	9.938	9.623	0.000
Intercept	4662.793	1	4662.793	4514.944	0.000
Online review inconsistency	22.156	1	22.156	21.454	0.000
Social distance	2.897	1	2.897	2.805	0.085
Online review inconsistency * Social distance	4.434	1	4.434	4.294	0.039
Error	289.169	280	1.033		
Total	4970.249	284			
Corrected total	318.983	283			

^a^*R*^2^ = 0.178 (Adjusted *R*^2^ = 0.159).

From the analysis of between-group variance in Table 1, the interaction effect between online review inconsistency and social distance is significant at the 0.05 level, *F*(1, 280) = 4.294, *p* = 0.039, indicating that social distance moderates the relationship between online review inconsistency and consumer purchase intentions. This study’s hypothesis H3 is supported. Following the aforementioned two-factor variance analysis results, this study needs to proceed with simple effects tests. In the simple effects tests, inconsistent and consistent online reviews are manipulated, aiming to dissect the moderating role of social distance in the formation of consumers’ online purchase intentions. The data for the simple effects analysis of this study are presented in Tables 2–4.

**Table 2:** Estimation of simple effects test for purchase intentions.

Online review inconsistency	Social distance	Means	Standard error	95%Confidence interval^b^	
				Lower limit	Upper limit
Inconsistent	Close	3.548	0.119	3.314	3.782
	Remote	4.000	0.120	3.764	4.236
Consistent	Close	4.357	0.121	4.119	4.594
	Remote	4.309	0.123	4.066	4.551

**Table 3:** Pairwise comparisons of simple effects tests on purchase intentions.

Online review inconsistency	(I) Social distance	(J) Social Distance	Mean difference (I-J)	Standar d error	Significance^b^	95% Confidence interval^b^	
						Lower limit	Upper limit
Inconsistent	Close	Remote	−0.452**	0.169	0.008	−0.784	−0.120
	Remote	Close	0.452**	0.169	0.008	0.120	0.784
Consistent	Close	Remote	0.048	0.172	0.781	−0.291	0.387
	Remote	Close	−0.048	0.172	0.781	−0.387	0.291

Based on estimated marginal means. ** Mean difference is significant at the 0.05 level. ^b^Multiple comparison adjustment: Sidak method.

**Table 4:** One-way analysis of simple effects on purchase intention.

Online review inconsistency		Sum of squares	Degrees of freedom	Mean square	*F*	Significance
Inconsistent	Contrast	7.407	1	7.407	7.173	0.008
	Error	289.169	280	1.033		
Consistent	Contrast	0.080	1	0.080	0.077	0.781
	Error	289.169	280	1.033		

When participants were exposed to situations of inconsistent online reviews, the main effect of social distance was significant at the 0.05 level, *F*(1, 280) = 7.173, *p* = 0.008. In this scenario, consumers’ purchase intentions (*M* = 3.548, SD = 0.119) in the close social distance condition were significantly lower than those of consumers in the remote social distance condition (*M* = 4.000, SD = 0.120), consistent with the hypothesis of this study, thus validating Hypothesis H6a. Conversely, when participants were exposed to consistent online review conditions, the main effect of social distance was not significant, *F*(1, 280) = 0.077, *p* = 0.781. In this case, the purchase intentions of consumers in the close social distance condition (*M* = 4.357, SD = 0.121) were slightly higher than those in the remote social distance condition (*M* = 4.309, SD = 0.123), but this difference was not statistically significant. This indicates that when consistent online reviews are matched with different levels of social distance, there is no significant variation in consumers’ purchase intentions, thus failing to support Hypothesis H3b. Combining the results of between-group variance analysis and simple effects tests, Hypotheses H3 and H3a are supported, while H3b is not supported and therefore rejected.

**Figure 3:**
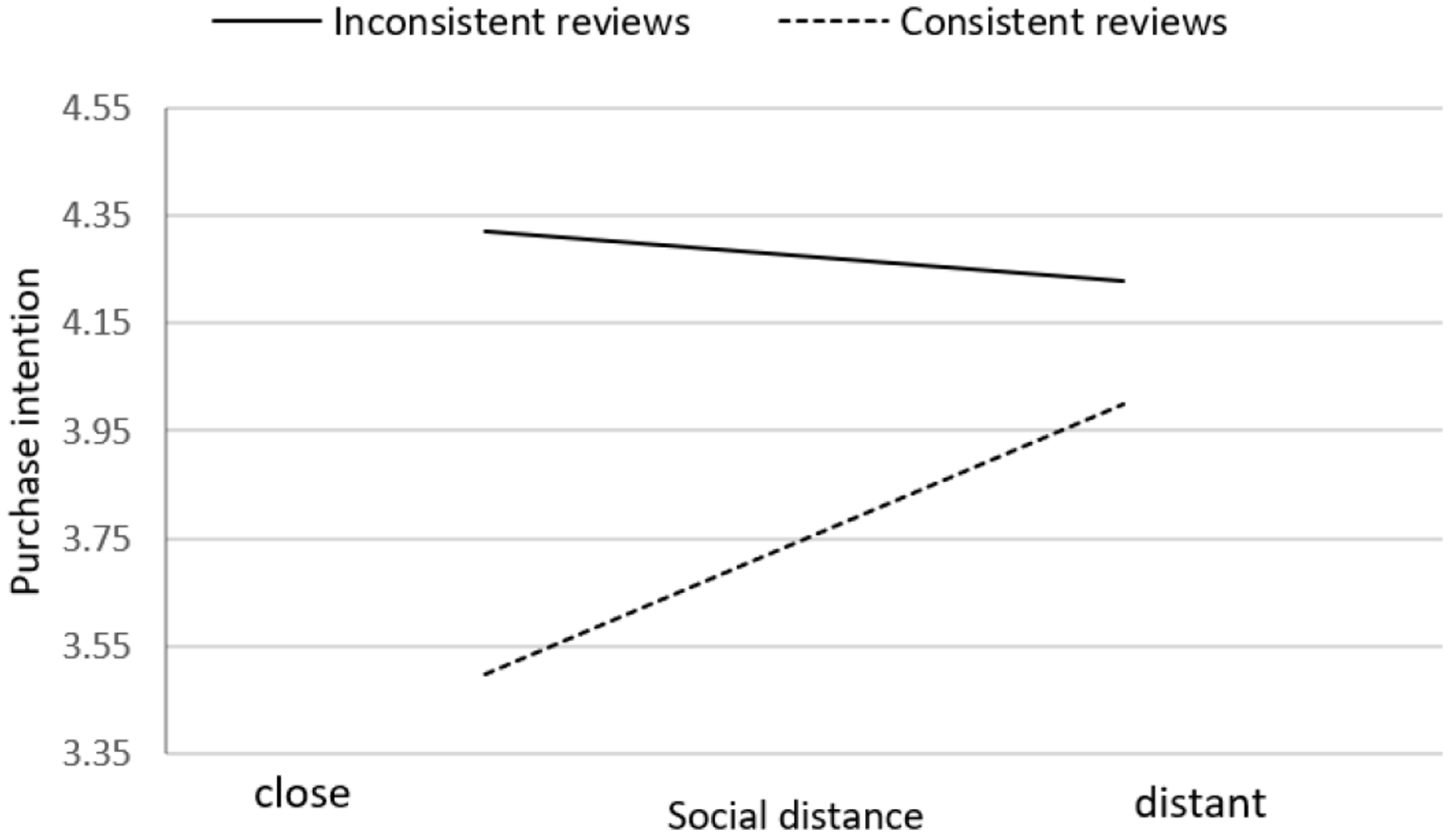
Line graph of consumer purchase intention under different levels of social distance.

Figure 3 illustrates consumer purchase intentions under varying social distances, in the context of both inconsistent and consistent online reviews. Facing inconsistent online reviews, purchase intentions increase as the remote social distance expands; however, in the case of consistent online reviews, even though the social distance increases, the change in consumer purchase intentions remains minimal. At the same social distance, consumers exposed to inconsistent online reviews exhibit lower purchase intentions compared to those exposed to consistent online reviews.

#### 3.1.3 Analysis

Study 1 employed a situational experimental method, which had high external validity. The sample consisted of college students from universities, which ensured high internal validity. The results showed that the inconsistency of online comments had a significant negative impact on consumers’ purchase intentions, and the conflicting attitude played a mediating role between the inconsistency of online comments and purchase intentions. We tested the moderating effect of social distance on the relationship between the inconsistency of online comments and consumers’ conflicting attitude and purchase intentions, respectively, under the conditions of inconsistent and consistent online comments.

The impact of the inconsistency of online comments on consumers’ purchase intentions varied under different social distances. The experimental results of Study 1 supported hypothesis H3a, indicating that when consumers encountered inconsistent online comments while shopping online, the conflicting attitude of consumers was significantly higher under the near social distance mode than under the far social distance mode. When consumers were in a far social distance, they would activate a higher level of interpretation when browsing products and online comments, and the negative information in the inconsistency of online comments had a lower impact on consumers than under the near social distance mode. This conclusion also verified that negative information had a lower impact on individuals as social distance increased compared to positive information. Therefore, consumers’ purchase intentions were significantly lower under the near social distance mode when encountering inconsistent online comments while shopping online than under the far social distance mode. Under the near social distance, consumers activated a lower level of interpretation, paying more attention to the specific details of the inconsistency of online comments, especially the negative information, which led to a lower purchase intention. Under the far social distance, consumers used a higher level of interpretation to perceive the consistency of online comments. The impact of negative information in the inconsistency of online comments was reduced, and consumers’ purchase intentions were higher than under the near social distance mode. This also explains why some gift products sold on e-commerce platforms have received many negative comments but have been selling well with increasing sales.

The experimental results of consumers encountering consistent online comments while shopping online did not support hypothesis H3b. This suggests that when online review information is consistent and clear, social distance may lose its moderating effect. Regarding the unsupported hypothesis, we analyzed the possible reasons. According to the certainty effect theory, consistent reviews may reduce the uncertainty in decision-making, thereby diminishing the impact of social distance. Previous research has shown that social distance can affect individuals’ risk perception (Lin, 2018). Under near social distance, individuals have a lower risk perception, and the impact of consistent online comments on consumers’ conflicting attitude is also small, which leads to no significant difference in the mean scores of consumers’ purchase intentions under the near social distance and far social distance modes when encountering consistent online comments.

### 3.2 Study 2

The study 2 used a 2 (Inconsistent online reviews: Inconsistent/Consistent) × 2 (Social distance: Close/Remote) × 2 (Product type: Experience/Search) design. On the one hand, it replicated the main effects and moderating role of psychological distance, as hypothesized in hypotheses 1, 2, and 3. On the other hand, it aimed to test the further moderation effect of product type on the psychological distance, as hypothesized in hypothesis 4. By doing so, this study aimed to contribute to the field of psychology by providing empirical evidence on the impact of inconsistency of online reviews, social distance, and type of product on consumers’ attitudes and behavior.

#### 3.2.1 Experimental process

In this experiment, two types of products were used: search products and experience products. Based on previous studies on the selection of search and experience products, the experiment was simplified by choosing a mobile phone as the search product when selecting products for online shopping (Zhang and Li, 2016). There are two main reasons for selecting a mobile phone as the search product. First, the participants in the experiment are university students who frequently use mobile phones and are familiar with them. Second, mobile phones are highly standardized products, and the various performance indicators can be clearly defined before consumers make a purchase, such as memory size, camera pixels, screen resolution, which can be presented in numerical forms. As for the selection of experience products, the experiment chose gourmet food (Zhang and Li, 2016) as the representative. Gourmet food is a product that consumers value for personal experience and preference characteristics. Consumers find it difficult to accurately know its core features and usually rely on information such as product promotion by e-commerce platforms (such as pictures, videos), online reviews (such as product quality, appearance, taste), and other information to make judgments. Only after purchase and consumption can consumers truly understand the information about gourmet food and assess its value (Akhtar et al., 2019; Li and Ren, 2017).

The raw data for the online reviews in Study 2 are sourced from two well-known e-commerce platforms: Taobao and Tripadvisor. Taobao is a prominent online retail platform and commercial hub in the Asia-Pacific region, with nearly 500 million registered users. It is a popular online shopping platform. Tripadvisor, on the other hand, is a leading global travel website that comprehensively covers hotels, attractions, restaurants, and airlines worldwide. It contains a significant amount of online review information specifically related to the restaurant industry (Wang et al., 2021). Therefore, the raw data for online reviews of mobile phones were sourced from Taobao. The raw data for online reviews of gourmet food were sourced from TripAdvisor.

For this study, we utilized a scenario-based simulation and recruited college students from a university in Heilongjiang province, China as our sample. A total of 566 participants were recruited in multiple batches through random selection. We conducted experiments involving eight different scenarios, with the manipulation of product types (experience/search) and other manipulations identical to Study 1. After the completion of the situational experiment, all participants will receive a high-quality souvenir worth $3.50 as a token of appreciation.

After collecting the questionnaires, we excluded incomplete responses and invalid questionnaires from participants who had never engaged in online shopping. Ultimately, a total of 551 valid questionnaires were collected, resulting in a response rate of 97.3%. The demographic analysis revealed that among the participants, there were 253 males, accounting for 45.9%, and 298 females, accounting for 54.1%, indicating that the gender ratio met the experimental requirements. In terms of online shopping experience, 78 participants reported having made online purchases a few times, while 395 participants indicated that they frequently made purchases online. Furthermore, 78 participants mentioned that they preferred to shop online whenever they had the opportunity. Approximately 85.8% of the participants reported having experienced online shopping frequently or always, reflecting a rich online shopping experience. Regarding disposable monthly income, 86 participants reported an income below 1,000 yuan, 339 participants reported an income between 1,001 and 2000 yuan, and 126 participants reported an income above 2001 yuan. In terms of online shopping duration, 46 participants reported less than 1 year of experience, 189 participants reported 1–3 years of experience, 241 participants reported 3–6 years of experience, and 75 participants reported more than 6 years of experience. The demographic characteristics of the sample aligned with the actual population, meeting the requirements of this study.

#### 3.2.2 Results

Inconsistency of Online Reviews: Manipulation tests revealed successful manipulation of perceived inconsistency of online reviews. Participants in the inconsistency condition (*M* = 3.8778, SD = 1.12843) perceived stronger inconsistency compared to participants in the consistency condition (*M* = 4.3895, SD = 1.10645) (*F* = 5.120, *p* = 0.000 < 0.05).

Social Distance: Statistical analysis was conducted to examine the difference in social distance between purchasing for closest friends and purchasing for ordinary classmates. Purchasing for closest friends was associated with a close social distance (*M* = 3.10, SD = 1.974), while purchasing for ordinary classmates was associated with a remote social distance (*M* = 4.50, SD = 1.346). The difference in social distance between the two groups was significant (*F* = 77.379, *p* = 0.000 < 0.05), indicating successful manipulation of social distance.

As shown in Table 5, our regression analysis once again reveals that the inconsistency of online reviews has an impact on consumers’ conflicting attitudes, which in turn affects their purchasing intentions. Specifically, the inconsistency of product reviews leads to an increase in consumers’ conflicting attitudes and a subsequent decrease in their willingness to purchase. Additionally, the analysis of between-group variances also found that the moderating effect of social distance remains significant. The three-factor analysis of variance with purchasing intention as the independent variable yielded the following results: the main effect of the inconsistency of online reviews is significant at the 0.05 level, *F*(1,543) = 15.158, *p* = 0.000; the main effect of product type is significant at the 0.05 level, *F*(1,543) = 6.042, *p* = 0.014. The interaction effect between the inconsistency of online reviews and social distance is significant at the 0.05 level, *F*(1,543) = 4.786, *p* = 0.029. Furthermore, the three-factor interaction effect between the inconsistency of online reviews, social distance, and product type is also significant at the 0.05 level, *F*(1,543) = 4.760, p = 0.030. These results demonstrate that product type further moderates the relationship between social distance, the inconsistency of online reviews, and consumers’ conflicting attitudes. Thus, this study provides support and validation for hypothesis H4.

**Table 5:** Results of three-way between-subjects ANOVA (dependent variable: purchase intention).

Source	III Type sum of squares	Degrees of freedom	Mean square	*F*	Significance
Corrected model	41.486^a^	7	5.927	4.785	0.000
Intercept	9242.405	1	9242.405	7461.463	0.000
Online review inconsistency	18.776	1	18.776	15.158	0.000
Social distance	7.484	1	7.484	6.042	0.014
Product type	1.857	1	1.857	1.499	0.221
Online review inconsistency * Social distance	5.929	1	5.929	4.786	0.029
Online review inconsistency * Product type	1.524	1	1.524	1.230	0.268
Social distance * Product type	0.072	1	0.072	0.058	0.809
Online review inconsistency * Social distance * Product type	5.830	1	5.830	4.706	0.030
Error	672.606	543	1.239		
Total	9951.000	551			
Corrected total	714.093	550			

^a^*R*^2^ = 0.198 (Adjusted *R*^2^ = 0.184).

Based on the results of the three-factor analysis of variance mentioned above, further *post hoc* tests are needed to examine the simple effects. In these post hoc tests, we manipulate the inconsistency of online reviews and social distance to analyze the role of product type in the relationship between social distance and consumers’ online purchasing intentions. Additionally, a classification analysis of the product type is conducted. The data for the post hoc tests in this study are presented in Tables 6–8.

**Table 6:** Estimated simple effects test of consumers’ purchase intention.

Online review inconsistency	Social distance	Product type	Means	Standard error	95% Confidence interval^b^	
					Lower limit	Upper limit
Inconsistent	Close	Search	3.674	0.131	3.416	3.931
		Experience	3.712	0.137	3.443	3.981
	Remote	Search	3.932	0.130	3.676	4.187
		Experience	4.336	0.136	4.069	4.603
Consistent	Close	Search	4.150	0.133	3.889	4.411
		Experience	4.390	0.135	4.125	4.655
	Remote	Search	4.404	0.135	4.139	4.670
		Experience	4.187	0.136	3.919	4.454

**Table 7:** Pairwise comparisons of simple effects tests on purchase intentions.

Online review inconsistency	Social distance	(M)Product type	(N)Product type	Means (M-N)	Standard error	Significance^b^	95%Confidence intervalb^b^	
							Lower limit	Upper limit
Inconsistent	Close	Search	Search	−0.039	0.190	0.839	−0.411	0.334
		Experience	Experience	0.039	0.190	0.839	−0.334	0.411
	Remote	Search	Search	−0.404**	0.188	0.032	−0.774	−0.034
		Experience	Experience	0.404**	0.188	0.032	0.034	0.774
Consistent	Close	Search	Search	−0.240	0.190	0.206	−0.612	0.133
		Experience	Experience	0.240	0.190	0.206	−0.133	0.612
	Remote	Search	Search	0.218	0.192	0.256	−0.158	0.594
		Experience	Experience	−0.218	0.192	0.256	−0.594	0.158

Based on estimated marginal means. **. Mean difference is significant at the 0.05 level. ^b^ Multiple comparison adjustment: Sidak method.

**Table 8:** One-way analysis of simple effects on purchase intention.

Online review inconsistency	Social distance		Sum of squares	Degrees of freedom	Mean square	*F*	Significance
Inconsistent	Close	Contrast	0.051	1	0.051	0.041	0.839
		Error	672.606	543	1.239		
	Remote	Contrast	5.711	1	5.711	4.610	0.032
		Error	672.606	543	1.239		
Consistent	Close	Contrast	1.982	1	1.982	1.600	0.206
		Error	672.606	543	1.239		
	Remote	Contrast	1.602	1	1.602	1.293	0.256
		Error	672.606	543	1.239		

In the context of inconsistent online reviews, the interaction effect between product type and remote social distance was found to be significant at the 0.05 level, *F*(1, 543) = 5.924, *p* = 0.016. Specifically, the purchase intention of participants for experience products (*M* = 4.336, SD = 0.136) was significantly higher than the purchase intention for search products (*M* = 3.932, SD = 0.130). This indicates that when consumers face inconsistent online reviews while shopping online and have a remote social distance, they are more inclined to have a stronger purchase intention for experience products compared to search products. However, the interaction effect between product type and close social distance was not significant, *F*(1, 543) = 0.041, *p* = 0.839. In this case, the purchase intention for experience products (*M* = 3.712, SD = 0.131) was higher than the purchase intention for search products (*M* = 3.674, SD = 0.131), but this difference was not statistically significant. Based on the above analysis, the hypothesis H4a of this study is partially supported.

In the context of consistent online reviews, the interaction effect between product type and remote social distance was not significant, *F*(1,543) = 1.293, *p* = 0.256. Specifically, the purchase intention for experience products (*M* = 4.187, SD = 0.136) was lower than the purchase intention for search products (*M* = 4.404, SD = 0.135), but this difference was not statistically significant. This suggests that when consumers face consistent online reviews and have a remote social distance, the difference in purchase intention for experience products (vs. search products) is not significant. Similarly, the interaction effect between product type and close social distance was not significant, *F*(1,543) = 1.600, *p* = 0.206. In this case, the purchase intention for experience products (*M* = 4.390, SD = 0.135) was higher than the purchase intention for search products (*M* = 4.150, SD = 0.133), but this difference was not statistically significant. This indicates that when consumers face consistent online reviews and have a close social distance, the difference in purchase intention for experience products (vs. search products) is not significant. Therefore, the hypothesis H4b is not supported.

**Figure 4:**
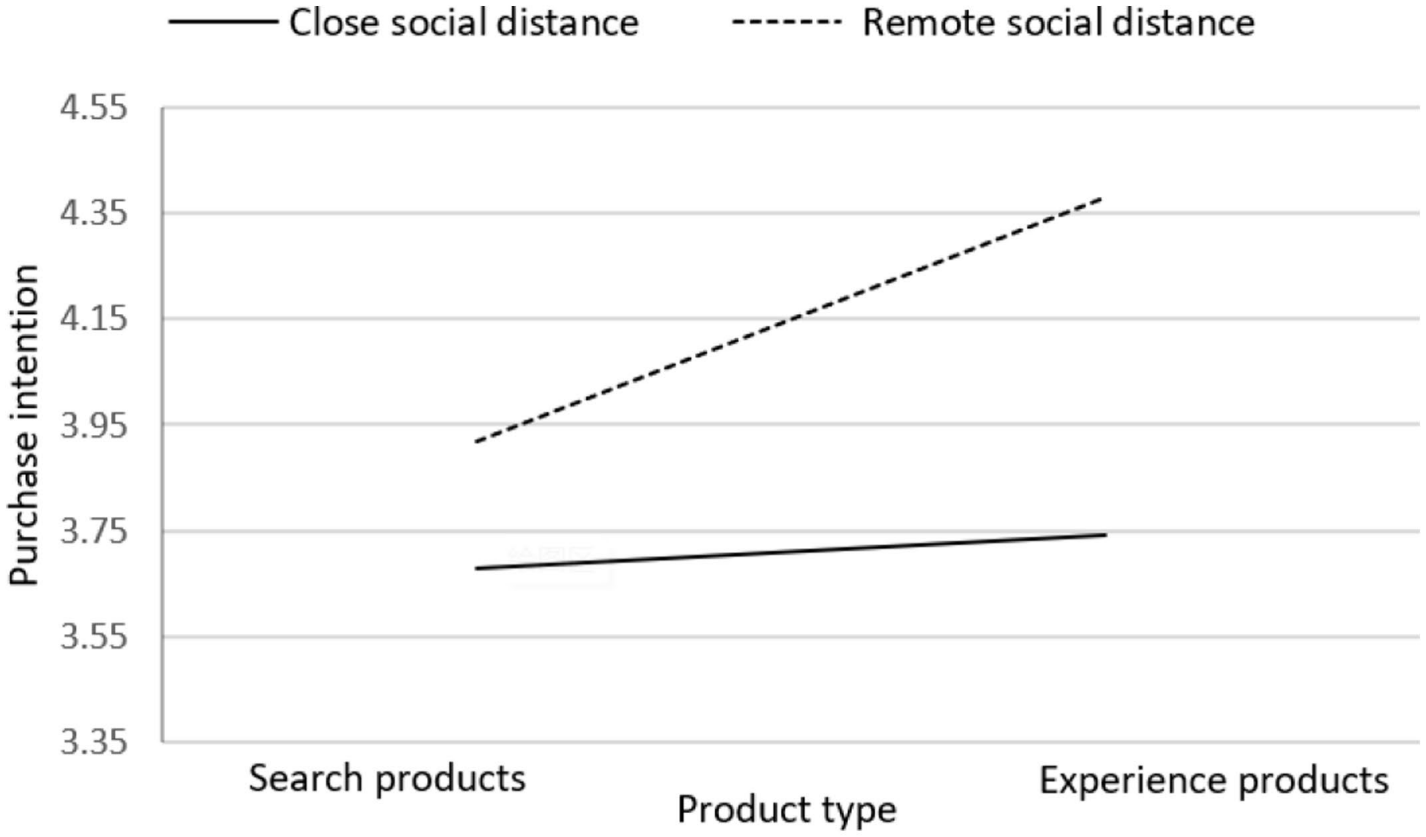
Line chart of consumers’ purchase intentions under different product types and social distance patterns in the context of inconsistent online reviews.

**Figure 5:**
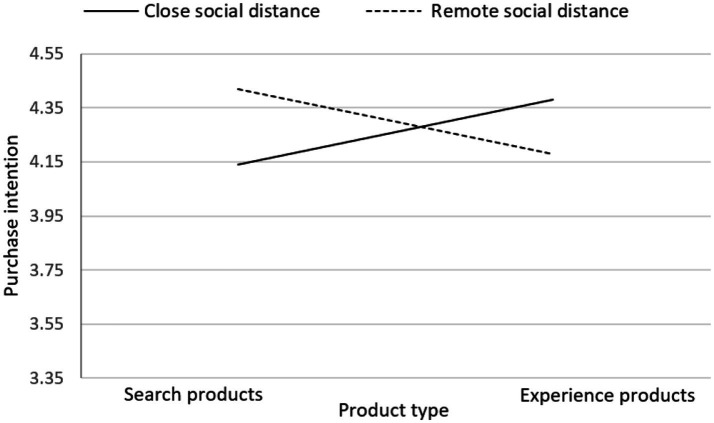
Line chart of consumers’ purchase intentions under different product types and social distance patterns in the context of consistent online reviews.

Figures 4, 5 present the changes in consumer purchase intention when facing inconsistent and consistent online reviews for both search products and experience products under different social distance modes. In the case of facing inconsistent online reviews, regardless of whether it is under remote social distance or close social distance mode, consumers show a higher purchase intention for experience products compared to search products. On the other hand, when facing consistent online reviews, under the remote social distance mode, consumers have a lower purchase intention for experience products compared to search products. Conversely, under the close social distance mode, consumers have a higher purchase intention for experience products compared to search products.

#### 3.2.3 Analysis

Study 2 incorporated product type (search/experience) and conducted a contextual experiment with three factors and eight conditions, examining the second-order moderation effect of product type on social distance. Study 2 was conducted strictly according to the experimental design and the results were generally as expected. The analysis of the experimental results revealed that the second-order moderation effect of product type exists within the theoretical framework of this study. Through the adjustment of social distance, it can influence the relationship between inconsistent online reviews and consumer ambivalence, further affecting purchase intention.

Study 1 has confirmed that there are differences in the impacts of inconsistent and consistent online reviews, which represent two forms of online comments, on consumer ambivalence and purchase intention. This finding demonstrates that the influence of social distance on purchase intention varies. Study 2 further examined the differential role of social distance in different types of products. The results of Study 2 revealed that when facing inconsistent online reviews in online shopping, consumers with remote social distance (purchasing for others) displayed significantly higher ambivalence toward experience products compared to search products. For consumers with close social distance (purchasing for individuals with whom they have a close relationship), there was no significant difference in ambivalence between purchasing search products and experience products. However, the mean scores indicate that consumers exhibited lower ambivalence when purchasing experience products compared to search products. Under conditions of remote social distance, consumers activate a higher level of cognitive processing to understand the inconsistency between online products and online comments. The negative impact of inconsistent online reviews on experience products was found to be more influential than on search products, which is consistent with the initial hypotheses. However, the experimental results did not support this conclusion under conditions of close social distance. This suggests that when consumers purchase products for individuals with whom they have close relationships, they tend to be more cautious and hesitant. A possible explanation is the influence of Chinese socio-cultural factors, as maintaining social relationships is highly valued in Chinese culture. However, this study did not empirically test the impact of cultural factors, and this hypothesis requires further research to explore this potential influence in greater depth. When facing consistent online reviews in online shopping, consumers with close social distance exhibited significantly higher ambivalence towards experience products compared to search products. Under conditions of remote social distance, there was no significant difference in ambivalence between purchasing search products and experience products. However, the mean scores suggest that consumers experienced higher ambivalence when purchasing experience products compared to search products, which aligns with their real-life experiences. Nonetheless, the experimental findings did not establish statistical significance for this observation.

In online shopping, when consumers encounter inconsistent online reviews, their purchase intentions for experiential products significantly exceed those for search products under conditions of remote social proximity. However, under close social proximity, there is no significant difference in purchase intentions between experiential and search products. Nonetheless, mean scores of ambivalent attitudes reveal that the purchase intention for experiential products remains higher than for search products.

Under remote social proximity, consumers engage higher levels of abstraction to cognitively process and understand online products and reviews. The influence of negative information in inconsistent reviews is diminished more in the context of experiential products than in search products, leading to stronger purchase intentions. However, this pattern does not hold when consumers face consistent online reviews. In scenarios of consistent reviews, regardless of social proximity, the difference in purchase intentions between experiential and search products is not significant. Consistent reviews have a minimal impact on consumer ambivalence across different product types and social distances, and consequently, a lesser effect on purchase intentions.

## 4 Conclusion

### 4.1 Theoretical implications

This study provides an in-depth analysis of the mediating role of ambivalence when the product recipient changes from oneself to others and investigates the mechanisms through which inconsistent online reviews influence consumer purchase intention. Specifically, it examines the mediating role of ambivalence, the moderating effect of social distance, and the second-order moderating effect of product type in the relationship between inconsistent online reviews and purchase intention. The study contributes to theory development in the following ways:

(1) Constructs a theoretical model of the influence mechanism of inconsistent online reviews on purchase intention with ambivalence as a mediator.

This research contributes to the theoretical understanding of how inconsistent online reviews influence consumer purchase intention by introducing ambivalence as a mediator in the process. It develops a comprehensive theoretical model that explains the intricate mechanisms through which inconsistent online reviews impact consumers’ decisions to purchase. By identifying ambivalence as a mediating factor, the study provides new insights into how conflicting information in reviews leads to mixed feelings, which in turn affects purchase intentions. Additionally, the model highlights the moderating role of social distance and its interaction with product type, showing how psychological factors and product characteristics influence the relationship between online review inconsistency and purchase behavior. This research enriches the literature on consumer decision-making and expands our understanding of the psychological processes involved in navigating inconsistent information in online reviews.

(2) Explains the mechanism of how inconsistent online reviews influence ambivalence and purchase intention under the moderation of social distance.

Existing literature on inconsistent online reviews has mostly focused on overall ratings (Chang et al., 2014) and review sources. [56] In contrast, this study examines the responses of consumers’ ambivalence and purchase intention to inconsistent online reviews from the perspective of social distance theory. It elucidates the underlying mechanisms through which inconsistent online reviews influence consumer purchase intention, which is the core focus of this research. By applying the concepts of psychological distance and construal level theory to the information processing of consumers facing inconsistent online reviews, the study provides an analysis and explanation of the underlying mechanisms. When consumers are at a remote social distance and making online purchases, they exhibit a higher level of construal and engage in more elaborate information processing when reading online reviews. The negative impact of inconsistent online reviews on consumers is found to be lower compared to when they are at a closer psychological distance. Interestingly, the influence of negative information decreases with the increase of temporal distance. Under high construal levels, individuals are more inclined to purchase the products they are facing in light of inconsistent online reviews. This research contributes to the integration of ambivalence theory and social distance theory in explaining the effects of inconsistent online reviews on consumers, providing a deeper understanding and application of these theoretical frameworks.

(3) Analyzes the mechanism of interaction between product type and social distance.

This study empirically examines the second-order moderating effect of product type, which further moderates the relationship between social distance and the influence of inconsistent online reviews on consumer purchase intention. It provides insights into the underlying relationship between inconsistent online reviews and consumer purchase intention and offers explanations for the contradictory findings in previous research. The study analyzes the impact of inconsistent online reviews on consumer ambivalence and purchase intention under different social distance scenarios, considering the moderating role of product type. The findings reveal that when consumers face inconsistent online reviews while shopping online and are at a remote social distance, their ambivalence is significantly higher for experiential products compared to search products. Moreover, consumers show a stronger purchase intention for experiential products compared to search products. This can be explained by the emphasis on interpersonal relationships and face-saving tendencies in Chinese culture, where consumers tend to be more cautious and considerate when shopping for individuals with closer social distance. In search products, inconsistent online reviews often highlight “product flaws,” which may lead to lower purchase intention for consumers at a remote social distance, even if their ambivalence is lower compared to consumers considering experiential products. This indicates a specific case in which construal level theory may not fully explain the mechanism of psychological distance. The study suggests that culture plays a significant role in influencing social distance dimensions and provides directions for future research. In summary, this study sheds light on the moderating effect of product type and social distance, contributing to a deeper understanding of the relationship between inconsistent online reviews and consumer behavior.

### 4.2 Managerial implications

This study provides valuable insights for online merchants and businesses by integrating the concept of social distance, a key component of psychological distance, into the decision-making process of consumers in response to the inconsistency of online reviews. Furthermore, it highlights the moderating role of product type on this mechanism. The findings carry specific managerial implications for enhancing consumer purchase intention and fostering a more objective understanding of review inconsistencies.

(1) Understanding the impact of inconsistent online reviews

Online merchants and businesses need to recognize the dual impact of inconsistent online reviews on consumer behavior. Our study reveals that while inconsistent reviews can significantly increase consumers’ ambivalent attitudes, they also reduce purchase intention. These effects can present substantial challenges to online sales, as previous research has shown (Tang et al., 2014; Ye et al., 2009). However, our findings indicate that the negative effects of review inconsistency can be moderated by psychological distance, which decreases as consumers perceive less psychological distance. This opens up new opportunities for businesses to adapt their strategies. For example, businesses should consider the psychological distance of different consumer segments and tailor their responses accordingly. For consumers who are undecided about their purchase, customer service can provide personalized advice, suggesting that they consider whether others in their social circle might need the product, thus framing the purchase as a gift. This approach helps reduce hesitation by reframing the purchase decision in terms of broader social relevance.

Additionally, the impact of product type on purchase decisions provides further strategic insights. Experience products (those consumers must use before evaluating) are less influenced by inconsistent reviews compared to search products (those whose quality can be assessed before purchase). Merchants selling search products should categorize their products based on the intended purpose and target the social distance preference of their audience. For example, if the product is likely to be purchased as a gift, marketing strategies could highlight its appeal to distant social connections. Conversely, for personal use products, which typically attract closer social connections, strategies can be tailored to emphasize their personal relevance.

(2) Helping consumers navigate review inconsistency

This study also offers guidance for consumers, helping them approach the inconsistency in online reviews more effectively. Given the moderating role of social distance, consumers should adopt a more nuanced perspective when interpreting inconsistent reviews based on the product type and their psychological distance from the product. Specifically, when purchasing experiential products (where the consumer’s experience is key to product evaluation), consumers should focus more on objectively analyzing review inconsistencies, especially when considering purchasing products for close relationships. This is because reviews of experiential products tend to reflect the subjective feelings of different consumers, and inconsistent reviews from different consumers do not necessarily indicate that the product is of poor quality. Consumers should understand that the differences in evaluations of experiential products are often closely related to individual preferences, needs, and usage environments. Therefore, when analyzing reviews, consumers should avoid simply viewing inconsistencies as a reflection of product quality and should instead consider the background and individual differences behind the reviews. In contrast, when purchasing search products, where consumers often rely on information about the product’s features and quality before buying, they should approach inconsistent reviews with caution, analyzing whether the inconsistencies are meaningful enough to reconsider or abandon the purchase. By considering both the product type and their psychological distance from the product, consumers can make more informed and rational decisions, which ultimately enhances their overall shopping experience.

### 4.3 Limitations and future research

This study has established a mechanistic model for examining the impact of inconsistent online reviews on consumer purchase intention, based on the perspective of psychological distance. It empirically tests the moderating role of social distance in the model and analyzes the second-order moderating effect of product type on social distance, which is of significance.

(1) In terms of questionnaire design, the experimental materials were sourced from a verified e-commerce platform to enhance the authenticity and reliability of the experimental context. However, there are still differences between experimental scenarios and actual online shopping environments, such as the quantity of reviews. When selecting experimental products, this study chose headphones as an online product that possesses characteristics of both search and experience products, with mobile phones representing search products and food representing experience products. However, other scholars have proposed that product types can also be further categorized into trust-based and non-trust-based (Hu et al., 2011). Therefore, whether the conclusions of this study are applicable to other products or different types of products requires further experimental verification, limiting the generalizability of the research findings. This study maintains a 1:1 ratio of positive and negative comments in the inconsistent online reviews, which allows for the inheritance of existing research and facilitates complementary comparisons (Ma and Hu, 2013). However, this manipulation differs from the actual ratio of positive and negative online reviews on e-commerce platforms.

(2) The study utilized a single sample of participants in each experiment. College students were chosen as participants primarily due to their high homogeneity, which reduces variability and the impact of irrelevant factors on experimental research, thereby enhancing internal validity. Additionally, college students have rich online shopping experiences and are representative of the main participants in scenario experiments. In future research, to enhance external validity, it would be beneficial to include diverse income groups and enrich the sample, making the experimental results more universally applicable.

(3) This study combined previous research and real-life practices and proposed that Chinese culture places significant emphasis on interpersonal relationships. It is speculated that cultural factors in China may be the reason for differences compared to previous research. The strong relationship culture in China may lead to cognitive deviations in perceiving the distance of social relationships that differ from other countries. Therefore, future research can incorporate cultural factors into the study of the moderating role of social distance.

## Data Availability

The raw data supporting the conclusions of this article will be made available by the authors, without undue reservation.
