# Supplementary material for: Shopping for others: how does inconsistency in online reviews affect purchase intentions differently?
Source: Front Psychol. 2025 Jun 19;16:1579545. doi: 10.3389/fpsyg.2025.1579545 (PMC12231527; doi:10.3389/fpsyg.2025.1579545)
Supplement: Supplementary file 1 [file Data_Sheet_1.docx]

Appendix 1: Social Distance Regulatory Role

(Close Social Distance Online Review Consensus Group)

Dear student:

Thank you for taking the time to participate in this survey. Please select the items in the questionnaire based on your genuine feelings. There are no right or wrong answers. The data collected will only be used for this study and is not related to any other commercial purposes. Please feel at ease to answer. Thank you for your participation, and we wish you academic success!

Please read the experimental instructions: Your closest friend's Bluetooth earphones recently broke, and you plan to purchase a new pair of Bluetooth earphones for him/her. On a shopping website, you noticed a specific brand of Bluetooth earphones. Please first review the graphic and textual information about this Bluetooth earphone.

1. Please select the options below based on the description you just read and your genuine thoughts.

|  | Very close  1 | 2 | 3 | 4 | 5 | 6 | Vary far  7 |
| --- | --- | --- | --- | --- | --- | --- | --- |
| You feel that the "closest friend" in the material, compared to an "ordinary classmate," is closer or farther from you in terms of distance. |  |  |  |  |  |  |  |

Before purchasing the Bluetooth earphones, you want to first learn about online reviews from consumers who have bought this product to make a decision. Please carefully read the relevant reviews and then select answers to the questionnaire questions.

Reviewer 1: The earphone sound quality is pure and clear, no noise, excellent audio quality, portable and easy to store, convenient connectivity, perfectly paired, very much liked. Wearing experience: Quite comfortable, no discomfort when inserted in the ears. Sound effect: Clear sound.

Reviewer 2: Wearing experience: Honestly different from ordinary earphones.Sound effect: Great sound quality, balanced left and right volume. Appearance/material: High-quality material, including the charging case.

Reviewer 3: Feels great for listening to music, no ear pressure. The earphones are simple and elegant, loud sound with no noise, excellent bass effect, clear sound quality, fast signal reception, very much liked.

Reviewer 4: The earphones have good quality, excellent sound effects, highly responsive touch controls, stable connection even in complex environments, overall good value for the price.

Reviewer 5: No issues after days of use, simple and convenient operation. Detailed sound quality, high sound reproduction accuracy, fully worth the price, no noise during music playback, excellent audio quality.

Reviewer 6:

Wearing experience: One of the best earphones I’ve worn, lightweight. Appearance/material: Sleek design, full premium feel. Sound effect: Good sound quality effects, excellent bass.

2. After reading the above reviews, please evaluate your perception of the review information.

|  | Strongly Disagree | Disagree | Somewhat Disagree | Neutral | Somewhat Agree | Agree | Strongly Agree |
| --- | --- | --- | --- | --- | --- | --- | --- |
| I believe the positive and negative information in the above reviews is consistent |  |  |  |  |  |  |  |
| I believe the opinions expressed in the above reviews are consistent |  |  |  |  |  |  |  |
| I believe the above review information is not contradictory |  |  |  |  |  |  |  |

3. Based on the online review information you just read, please select the following options according to your genuine thoughts:

|  | Strongly Disagree | Disagree | Somewhat Disagree | Neutral | Somewhat Agree | Agree | Strongly Agree |
| --- | --- | --- | --- | --- | --- | --- | --- |
| Purchasing this product makes me feel conflicted |  |  |  |  |  |  |  |
| Purchasing this product makes me feel mixed emotions |  |  |  |  |  |  |  |
| Purchasing this product makes me feel hesitant |  |  |  |  |  |  |  |

4. Based on the product and online review information you just read as the primary basis for your purchasing decision, please select the following options according to your genuine thoughts:

|  | Strongly Disagree | Disagree | Somewhat Disagree | Neutral | Somewhat Agree | Agree | Strongly Agree |
| --- | --- | --- | --- | --- | --- | --- | --- |
| I would consider purchasing this product |  |  |  |  |  |  |  |
| There is a possibility I would purchase this product |  |  |  |  |  |  |  |
| My intention to purchase this product is very strong |  |  |  |  |  |  |  |

5. Please answer based on your actual situation:

(1) Your gender: [Single choice]

○ Male ○ Female

(2) Your online shopping experience is: [Single choice]

○ Never shopped online ○ Shopped a few times ○ Shop frequently ○ Buy everything online

(3) Your monthly disposable income (for students, includes parental support, scholarships, grants, part-time income, etc.): [Single choice]

○ Below ¥1,000	○ ¥1,001 - ¥2,000	○ Above ¥2,001

(4) How long have you been shopping online? [Single choice]

○ Less than 1 year (excluding 1 year) ○ 1 - 3 years (excluding 3 years) ○ 3 - 6 years (excluding 6 years) ○ 6 years or more (including 6 years)

The experiment is now concluded. Thank you very much for your participation and assi
